# Down-Regulation Ovulation-Induction Leads to Favorable Outcomes in a Single Frozen-Thawed Blastocyst Transfer RCT

**DOI:** 10.3389/fendo.2022.797121

**Published:** 2022-03-07

**Authors:** Shi-Bin Chao, Yan-Hong Wang, Jian-Chun Li, Wen-Ting Cao, Yun Zhou, Qing-Yuan Sun

**Affiliations:** ^1^ Department of Clinical Medicine, Fuzhou Medical College of Nanchang University, Fuzhou, China; ^2^ ART Centre, Maternal and Child Health Care Hospital, Shangrao, China; ^3^ Department of Clinical Laboratory, The First Affiliated Hospital of Nanchang University, Nanchang, China; ^4^ Fertility Preservation Lab, Guangdong-Hong Kong Metabolism and Reproduction Joint Laboratory, Reproductive Medicine Centre, Guangdong Second Provincial General Hospital, Guangzhou, China

**Keywords:** down-regulation ovulation-induction, elective single embryo transfer, frozen-thawed embryo transfer, endometrial receptivity, endometrial preparation

## Abstract

**Objective:**

Elective single embryo transfer (eSET) has been increasingly advocated to achieve the goal of delivering a single healthy baby. A novel endometrial preparation approach down-regulation ovulation-induction (DROI) proposed by our team was demonstrated in an RCT that DROI could significantly improve the reproductive outcome compared with modified natural cycle. We aimed to evaluate whether DROI improved clinic pregnancy rate in this single frozen-thawed blastocyst transfer RCT compared with hormone replace treatment (HRT).

**Method:**

Eligible participants were recruited and randomized into one of two endometrial preparation regimens: DROI or HRT between March 15, 2019 and March 12, 2021. The primary outcome was clinical pregnancy rate (CPR). The secondary endpoints included ongoing pregnancy rate (OPR), biochemical miscarriage and first trimester pregnancy loss. This trial is registered at the Chinese Clinical Trial Registry, number ChiCTR2000039804.

**Result (s):**

A total of 330 women were randomized in a 1:1 ratio between two groups and 289 women received embryo transfer and completed the study (142 DROI; 147HRT). Pregnancy outcomes were significantly different between the two groups. The CPR and OPR in the DROI group were significantly higher than those of the HRT group (64.08% versus 46.94%, P<0.01; 56.34% versus 38.78%,P<0.01). The biochemical miscarriage and first trimester pregnancy loss were comparable between the two groups.

**Conclusion (s):**

The findings of this RCT support the suggestion that the DROI might be a more efficient and promising alternative endometrial preparation approach for FET. Moreover, DROI could play a critical role in promoting uptake of single embryo transfer strategies in FET.

## Introduction

Due to the well-known advantages of frozen-thawed embryo transfer (FET), such as prevention of ovarian hyperstimulation syndrome (OHSS) and increasing cumulative live birth rates (CLBRs) (1, 2), the proportion and the number of FET cycles performed have increased dramatically in recent years (3). In contrast to the complex COS, FET procedures are simpler, with the primary objective being to adequate preparation of the endometrium to receive the thawed embryo(s). Various endometrial preparation regimens have been developed for FET. However, there is no consensus on the optimal approach for endometrial preparation (4).

Multiple gestations are iatrogenic complications during IVF and bring a series of negative maternal and infant complications such as premature birth and low birth weight (5, 6). Elective single embryo transfer (eSET) is the most efficient approach to reduce the risk of multiple gestations (7). Despite strong advocacy for its universal adoption, its widespread uptake is slow because reducing the number of embryos transferred compromise the pregnancy rate (8). Many efforts have been made to increase the pregnancy rate after eSET (9). Extending embryo culture to blastocyst from cleavage stage allows for better evaluation of the implantation potential of the embryo (10). As a result, single blastocyst transfer strategy is recommended (11).

Whether in fresh ET or FET, successful implantation involves interactions between the endometrium and the embryo. It is estimated that embryos account for 30% of implantation failures, while suboptimal endometrial receptivity and altered embryo–endometrial dialogue are responsible for the remaining 70% (12, 13). However, embryo quality has been the most focused aspect of IVF over the past four decades while more and more studies are paying attention to endometrial receptivity (14, 15).

Inspired by preferable endometrial receptivity of the depot GnRH-a COS protocol (16, 17), we proposed the DROI as an endometrial preparation protocol and demonstrated in a pilot RCT that DROI could significantly improve the reproductive outcome of FET compared to modified natural cycle (18). In the previous pilot RCT, as the average number of embryos transferred in DROI group reached 1.67, the multiple pregnancy rates reached 30.28% that was unacceptable in modern IVF practice. Therefore, we designed this single blastocyst transfer RCT study. The main objective of this study was to demonstrate whether DROI regimen can improve the pregnancy outcome of single blastocyst FET compared with HRT.

## Materials and Methods

### Study Design and Participants

This study was conducted as a randomized clinical trial at the ART centre of Shangrao Maternal and Child Health Care Hospital, Jiangxi, China. The study conformed to the ‘Declaration of Helsinki for Medical Research involving Human Subjects’ and was approved by ethics committees of the hospital. This RCT trial was registered at the Chinese Clinical Trial Registry (ChiCTR2000039804). The candidate patients obtained detailed information of both approaches, including the duration of the down-regulation and the potential risk of pituitary suppression. All the couples gave written informed consent for the procedures.

Participants scheduled for FET were randomly assigned to two study groups in a 1:1 ratio. Random allocation was performed by a study doctor at endometrial preparation by means of computer-generated random numbers in sealed, unlabelled envelopes. This trial included women who had at least one blastocyst cryopreserved and were undergoing their first FET cycle. Other inclusion criteria were aged 20–40 years at the time their embryos were frozen, BMI 18–28 kg/m2, and basal FSH level<10 IU/ml. Women with hyperprolactinemia, endometriosis, hydrosalpinx and uterine abnormalities, thyroid disease were excluded from this study.

### COS and Embryo Vitrification, Thawing, and Transfer

All participants were given gonadotropin releasing hormone (GnRH) antagonist or Progestin-primed ovarian stimulation (PPOS) regimen for ovarian stimulation as extensively described elsewhere (19, 20). When at least two leading follicles were18 mm or greater in mean diameter, human chorionic gonadotropin (HCG) at a dose of 4000–10 000 IU or 0.2 mg of triptorelin was administered to trigger ovulation. Oocytes were retrieved transvaginally 35–37 h after trigger. Embryo morphology was assessed and graded on Day 3according to the Cummins criteria (21). Generally, two Grade I or Grade II embryos were vitrified or transferred on Day 3, and the surplus embryos were cultured to Day 5 or 6 to reach blastocyst stage for later FET cycles. The vitrification procedure was performed following standard protocols using Kitazato Freeze Kit (Kitazato Corporation, Japan). Blastocysts were graded according to the Gardner criteria, based on the expansion of the blastocoel cavity, number of cells and cohesiveness of the inner cell mass and trophectoderm (22). Blastocysts graded ≧4BB according to Gardner morphological criteria were classified as good-quality embryo.

### Endometrial Preparation Before Embryo Transfer

The procedure of DROI protocol was summarized in our previous report (18). Briefly, 75–150 IU of HMG (Lizhu Pharmaceutical Trading Co, Zhuhai, China) started after 35-42 Day administration of a full dose (3.75 mg) Leuprorelin Acetate (Lizhu Pharmaceutical Trading Co, Shanghai, China). Gonadotropin stimulation continued until endometrial thickness ≥7 mm and met one of the following two criteria (1): If there were dominant follicles, the number of leading follicles that had a mean diameter of ≥16 mm was between 1-3, with serum estradiol levels 200-800ng/l and progesterone<1.5ng/ml (2); If there was no dominant follicle, at most four follicles reached the diameter between 12-15mm, with serum estradiol levels between 150-1000ng/l and progesterone<1.5ng/ml. A dose of HCG 5000-10000 IU was injected at 9:00 PM, and ET was arranged 7 days later. Oral 30mg/d DYG (Dupbaston, Abbott, Netherlands) as luteal support was initiated 2 days after HCG trigger and continued to 6 weeks of gestation if a pregnancy occurred.

In HRT protocols, oral estradiol valerate (Progynova; Bayer, Germany) was commenced on the 2nd or 3rd day of a natural or progesterone-induced menstrual cycle, and 10–12 days later, ultrasound examination was carried out to measure endometrial thickness. When the endometrial thickness attained ≥7 mm, oral 20mg/d DYG (Dupbaston, Abbott, Netherlands) and 90 mg daily progesterone vaginal gel (Crinone, MERCK, UK) were initiated. Embryo transfer was performed 5 days later. Luteal support was continued to 10 weeks of gestation if a pregnancy occurred.

### Sample Size Calculation and Statistical Analysis

The CPR after single blastocyst transfer was about 50% in our retrospective clinical database. We assumed that an absolute difference of 15% in CPR from our pilot trial and thus aimed to test a difference of 15% of CPR between two groups at a significance level of 0.05 with statistical power of 80%. The minimal sample size was 132 for each group as calculated by PASS 11.0 software (https://www.ncss.com/software/pass/). In consideration of a cancellation rate of 20%, we planned to enroll 165 women in each group.

The primary outcome was analyzed according to the intention-to-treat principle. The difference in the primary outcome (CPR) and other categoric variables between the two treatment groups was analyzed by the Pearson χ² test. Continuous data were compared with the Student’s t-test. All statistical analyses were performed by using the Statistical Package for Social Sciences (SPSS) version 18.0. A P value <0.05 was considered to be statistically significant.

## Results

### Study Population

**Figure 1:**
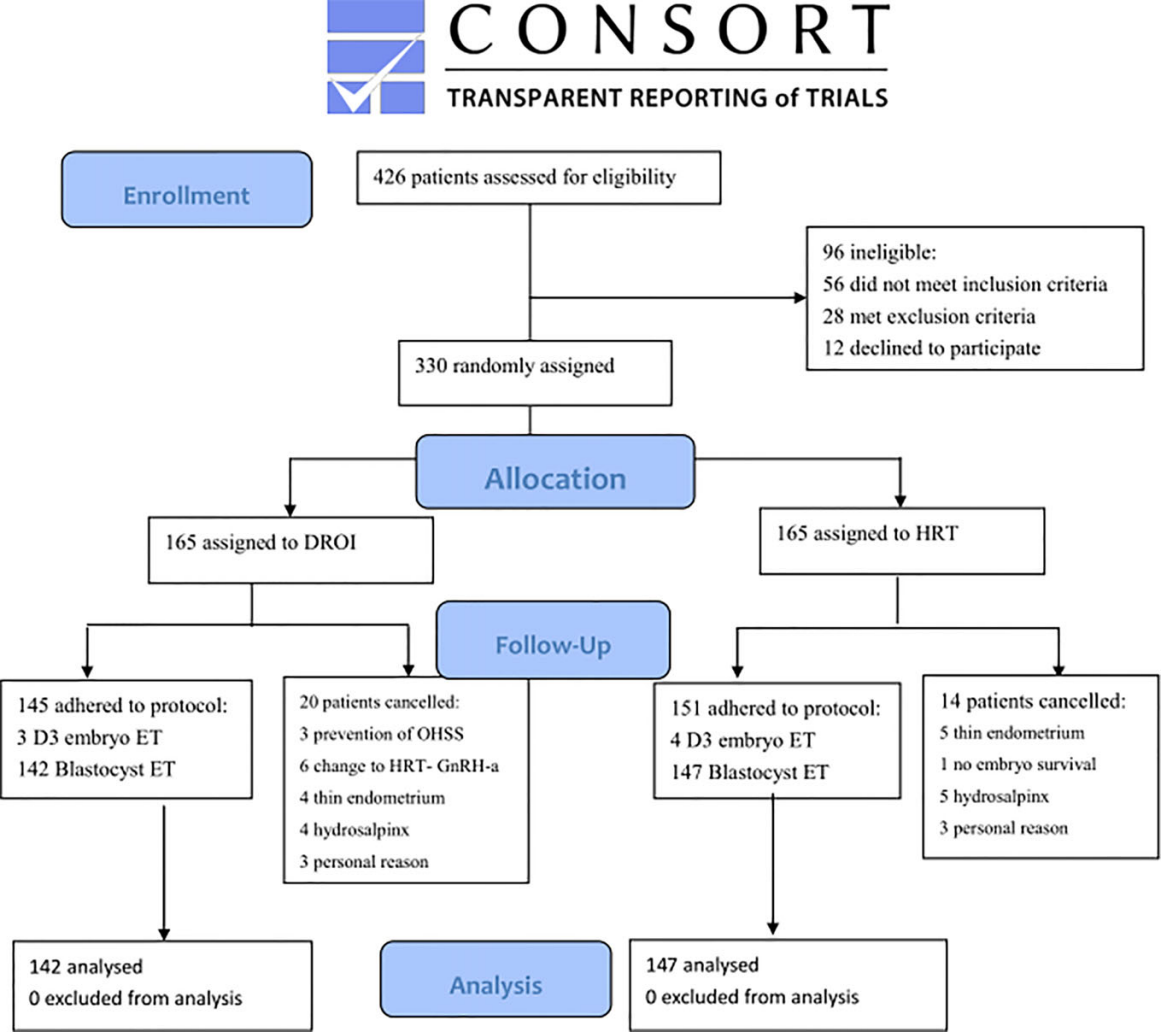
Flowchart showing of enrollment and randomization of study patients.

Recruitment was done between March 15, 2019, and March 12, 2021. A total of 330 women were randomized and 289 had FET eventually and completed the study. Reasons for dropout are summarized in **Figure 1**. Remaining patients received treatment according to study group allocation, resulting in 142 patients (49.13%) receiving DROI-FET and 147 (50.87%) receiving HRT-FET.

### Baseline Characteristics

Patients’ baseline demographics and clinical characteristics are detailed in **Table 1**. No significant difference was observed between the two treatment groups regarding age, BMI, AMH, duration of infertility, total antral follicle count, indication for IVF, or type of infertility. The baseline hormone profiles and COS protocols proportion in fresh cycles were similar between the two groups.

**Table 1:** Basic characteristics of patients at the cycle level.

Characteristic	DROI (142)	HRT (147)	P value
**Age (y)**	30.03 ± 4.51	29.82 ± 4.34	0.68
**Body mass index (kg/m2)**	22.07 ± 2.59	21.67 ± 2.33	0.17
**Infertility duration (years)**	3.17 ± 2.28	3.02 ± 2.55	0.61
**Type of infertility**			
**primary**	60 (42.25%)	62 (42.18%)	
**secondary**	82 (57.75%)	85 (57.82%)	0.99
**Indications for IVF**			
**Tubal factor**	88 (61.97%)	82 (55.78%)	
**Male factor**	27 (19.01%)	28 (19.05%)	
**Unexplained infertility**	5 (3.52%)	6 (4.08%)	
**Others**	22 (15.49%)	31 (21.09%)	0.62
**Baseline sex hormone**			
**FSH (IU/L)**	6.46 ± 1.63	6.62 ± 1.47	0.37
**LH (IU/L)**	5.05 ± 2.89	4.95 ± 2.56	0.74
**E2 (pg/mL)**	35.32 ± 10.43	34.38 ± 11.27	0.47
**AMH**	4.27 ± 2.76	4.89 ± 3.20	0.08
**Total antral follicle count**	15.09 ± 5.83	16.24 ± 6.11	0.10
**protocol for cos**			
**GnRH-ANT**	122 (85.92%)	115 (78.23%)	
**PPOS**	20 (14.02%)	32 (21.77%)	0.09

Data are presented as mean±SD for continuous variables and n (%) for dichotomous variables. All P values were assessed with the use of χ2 or Student t test. DROI, down-regulation ovulation-induction; HRT, hormone replacement treatment; AMH, anti-Müllerian hormone; PPOS, Progestin-primed ovarian stimulation.

### Cycle Characteristics of FET

The quality and developmental stage of blastocysts are the key factors that affect the outcome of FET. As presented in **Table 2**, the proportions of patients with good/poor quality and D5/D6 blastocysts transferred between the two groups were comparable. The expansion of the blastocoel cavity is also an important aspect of blastocyst quality. The majority of blastocysts transferred in this study were at stage 4 or 5 (96.48% VS 98.63%), and only a small number of stage 3 and 6 blastocysts were found in each group. The proportion of 3-6 stage expansion of the blastocoel cavity was not statistically significant between the two groups. The endometrium thickness on embryo transfer day was slightly thicker in the DROI group than in the HRT group (11.37 VS 10.96), but there was no statistical significance.

**Table 2:** Cycle characteristics at transfer level.

Characteristic	DROI (142)	HRT (147)	P value
**Blastocyst quality**			
**good**	133 (93.67%)	139 (94.56%)	
**poor**	9 (6.33%)	8 (5.44%)	0.75
**Expansion of the blastocoel cavity**			
**3**	1 (0.70%)	1 (0.68%)	
**4**	89 (62.68%)	90 (61.22%)	
**5**	48 (33.80%)	55 (37.41%)	
**6**	4 (2.81%)	1 (0.68%)	0.80
**Blastocyst stage**			
**D5**	115 (80.99%)	113 (76.87%)	
**D6**	27 (19.01%)	34 (23.13%)	0.39
**Endometrium thickness (mm)**	11.37±2.53	10.96±2.68	0.19

Data are presented as n (%) for dichotomous variables. All P values were assessed with the use of χ2. Abbreviations as in **Table 1**.

### Reproductive Outcomes of FET

The main reproductive outcomes of FET are presented in **Table 3**. Our primary outcome, the clinical pregnancy rate in the DROI group, was statistically higher than that in the HRT group (64.08% versus 46.94%, P<0.01). The rate of ongoing pregnancy in the DROI group was also higher than that in the HRT group (56.34% versus 38.78%, P<0.01). The biochemical miscarriage and first trimester pregnancy loss were comparable between two groups (11.26% versus 11.56%, P>0.05; 9.89% versus 15.94%, P>0.05). There were 2 cases of ectopic pregnancy in the DROI group and 1 case in the HRT group.

**Table 3:** Reproductive outcomes.

Characteristic	DROI(142)	HRT(147)	P value
**Biochemical miscarriage (%)**	16 (11.26%)	17 (11.56%)	0.94
**Clinical pregnancy (%)**	91 (64.08%)	69 (46.94%)	<0.01
**Ectopic pregnancy (%)**	2 (1.41%)	1 (0.68%)	0.98
**First trimester pregnancyLoss (%)**	9 (9.89%)	11 (15.94%)	0.70
**Ongoing pregnancy (%)**	80 (56.34%)	57 (38.78%)	<0.01

Data are presented as n (%) for dichotomous variables. All P values were assessed with the use of χ2. Biochemical miscarriage: serum hCG testing over 50IU/L on 14th day after ET but not confirmed clinical pregnancy; Clinical pregnancy: detection of at least one gestational sac in the uterine cavity on ultrasound at 4 weeks after ET; Ectopic pregnancy: observation of a gestational sac outside uterine cavity via ultrasound; First trimester pregnancy loss: spontaneous pregnancy loss less than 12 weeks of gestation after clinical pregnant; Ongoing pregnancy: detection of a viable fetus with fetal heartbeat at 12 weeks’ gestation.

## Discussion

To the best of our knowledge, Down-regulation ovulation-induction is a novel endometrial preparation protocol first proposed and practiced in FET by our team (18). The results of this study showed that the DROI approach could significantly improve CPR and OPR in single blastocyst FET (64.08% VS 46.94%, P<0.01; 56.34% VS 38.78%, P<0.01). We assumed that the underlying mechanism for this better pregnancy outcome is favorable endometrial receptivity. Since Rock and Bartlett described the histological changes of the endometrium around the time of implantation (23), many studies and efforts have been made to improve endometrial receptivity, but there is little consensus (13). Depot GnRH-a protocol has been widely used in China and it dramatically improved reproductive outcomes (16, 24). The success of the protocol proved to be due to improved endometrial receptivity (17, 25). DROI mimics the depot GnRH-a regimen procedure to take advantage of its favorable endometrial receptivity. Prolongation of downregulation increased the expression of the enzymes and cytokines directly and promoted the level of endometrial receptivity markers such as integrin-b3 and leukemia inhibitory factors (26–28). The combination of endogenous estrogen, progesterone, and HCG trigger in DROI regimen might promote endometrial receptivity marker expression by some mechanism that we don’t know yet.

In recent years, FET has become more widely used and played an increasingly important role in IVF. Therefore, the quality of FET is a key element of IVF quality. Endometrial preparation is the most critical step for FET. The options range from natural cycle, over ovarian stimulation, to HRT with or without GnRH-a (1). Indeed, in the latest review, it was concluded that no regimen was superior to another in relation to reproductive outcomes (29, 30). A more effective approach is highly desirable for doctors and patients. In the previous and present studies, we have shown that DROI outperforms both mNC and HRT.

The goal of all fertility treatments is the delivery of a single healthy baby. In the early days of IVF, the guidelines involved the transfer of multiple embryos to achieve a relatively acceptable CPR. High multiple birth rates present a substantial problem and have consequently poorer obstetric and neonatal outcomes. ESET was proposed as the best method to reduce multiple gestations (31). The reduction in the number of embryos transferred has also resulted in a reduction in CPR, thus limiting the uptake of eSET strategies (32–34). Strategies to screen out embryos with the best developmental potential seem ineffective (35–37). Improving endometrial receptivity may be a more promising approach. The favorable outcomes of DROI in this RCT may be a beneficial effect on endometrial receptivity.

There are limitations in this study that should be taken into account when interpreting the findings. First, it is a small sample size, single-centre study which may be subject to selection bias. Second, participants recruited for this RCT were patients with a good prognosis. We should be cautious to generalize the outcomes to women with an unfavorable or even less favorable prognosis. Third, live birth rate and obstetric and perinatal complications are not yet presented in the study. Larger sample size, multi-centre RCT and a more detailed study are needed to verify the practicability of the protocol in future studies.

In conclusion, DROI showed significant advantages in single frozen-thawed blastocyst transfer over HRT. A possible explanation for the better outcomes with the DROI may benefit effect on endometrial receptivity as the depot GnRH-a COS protocol. Therefore, DROI would be a more effective and promising alternative endometrial preparation regimen and might play an irreplaceable role in promoting uptake eSET strategy in FET.

## Data Availability Statement

The raw data supporting the conclusions of this article will be made available by the authors, without undue reservation.

## Ethics Statement

The studies involving human participants were reviewed and approved by Ethics committees of ShangRao Maternal and Child Health Care Hospital. The patients/participants provided their written informed consent to participate in this study.

## Author Contributions

S-BC, conception and design of experiments, acquisition of data, analysis and interpretation of data, writing and revising the article. Y-HW, YZ, J-CL, acquisition of data, analysis and interpretation of data. W-TC, revising the article. Q-YS, conception and design of experiments, revising the article. All authors contributed to the article and approved the submitted version.

## Funding

This study was supported by Key Research and Development projects of Jiangxi Province, China (NO: 20171BBG70010, 20192BBG70005).

## Conflict of Interest

The authors declare that the research was conducted in the absence of any commercial or financial relationships that could be construed as a potential conflict of interest.

## Publisher’s Note

All claims expressed in this article are solely those of the authors and do not necessarily represent those of their affiliated organizations, or those of the publisher, the editors and the reviewers. Any product that may be evaluated in this article, or claim that may be made by its manufacturer, is not guaranteed or endorsed by the publisher.
